# Genomic analysis and characterization of lytic bacteriophages that target antimicrobial resistant *Escherichia coli* in Addis Ababa, Ethiopia

**DOI:** 10.1016/j.heliyon.2024.e40342

**Published:** 2024-11-12

**Authors:** Tamirat Salile Sada, Dawit Hailu Alemayehu, Kalkidan Melaku Tafese, Tesfaye Sisay Tessema

**Affiliations:** a Institute of Biotechnology, Addis Ababa University, Addis Ababa, Ethiopia; b Department of Biotechnology, Woldia University, North Wollo, Woldia, Ethiopia; c Virology division, Armauer Hansen Research Institute, ALERT Center, Addis Ababa, Ethiopia; d Bacteriology division, Armauer Hansen Research Institute, ALERT Center, Addis Ababa, Ethiopia

**Keywords:** Antibiotic resistance, *Caudoviricetes*, *Escherichia coli*, Genome sequencing, Isolates, Lytic bacteriophage, Phage therapy

## Abstract

The emergence of antibiotic resistance in *E. coli* strains has sparked a fervent investigation of alternative therapies, such as the use of lytic bacteriophages. Phage genome sequence analysis is a novel method for learning more about proteins and other biomolecules encoded by phages, particularly phage-lytic enzymes that are crucial to the lysis of bacterial cells. Seven potential lytic E. coli phages—EH-B-A (A1), EP-M-A, EP-B-K (E2), EI-SP-GF, ET-SD-TH, and ST-TK—isolated from activated dairy farm sludges, rivers, and hospital liquid waste genomes were described in this study. The Illumina NextSeq 550 sequencer was used for sequencing phage isolates. The virus nucleotide collection (nr/nt) (taxid:10239) was used to evaluate whole-genome sequences. Phylogenetic analysis was performed using the MEGA11 software. Genome sequencing revealed that each bacteriophage contained a linear double-stranded DNA genome. Phage isolates were taxonomically identified as the *Caudoviricetes* class with four genera of phages, including *Kagunavirus, Vequintavirus, Dhillonvirus*, and *Jilinvirus*. Phage genome lengths varied from 24,264 to 136,204 bp, and GC contents ranged from 44 % to 55 %. In total, 33–218 CDSs (coding sequences) were predicted, with 19–77 % of CDSs encoding functional proteins. All phages lacked tRNA genes in their genomes, except for EI-SP-GF, which possessed five tRNA genes. Based on the phylogenetic tree analysis, the phage isolates were related to *Enterobacteria* and *E. coli* phage sequences in the database. Screening did not show any genes encoding for a CRISPR-like system, virulence, antibiotic resistance, or lysogeny. Because of their stringent lytic nature, these phage isolates might be used in the future to treat *E. coli* infections. This study might also provide some primary data for developing phage control techniques and advance our understanding of the genetic composition of *E. coli* phages.

## Highlights

• The use of antibiotics to treat *E. coli* infections often leads to the dissemination of multidrug resistance (MDR) in veterinary and clinical settings, and henece bacteriophages become a promising alternatives.
• Phage genome sequencing is a novel method for learning more about genome characteristics, gene functions, genome-based taxonomy, and relationship analysis as well as sequence information provides details about proteins and other biomolecules encoded by phages, such as phage lytic enzymes.
• Lytic *Caudoviricete* phages are good candidate types of phages used as an alternative therapy for multi-drug resistant pathogenic *E. coli.*
• The genome of lytic diarrheagenic coliphages has linear double-stranded DNA that was evolutionarily related to *E. coli* and *Enterobacteria* phages in the database.
• Since phages lack resistance and lysogenic genes as well as contain potential lytic enzymes, they were intended to combat different strains of multidrug-resistant diarrheagenic *E. coli* strains that are frequently associated with infections in humans and animals.

## 1 Background

*Escherichia coli* (*E. coli*) includes both commensal and pathogenic strains, mostly harboring human and animal intestines, and it is possibly the most widely studied bacterial species. It can also be present in food, vegetation, sewerage, and the environment. While most *E. coli* strains are safe and contribute to the normal gut flora, some strains are harmful and can cause serious illness or food poisoning. *E. coli* is the most prevalent causal agent of urinary tract infections (UTIs) worldwide, accounting for 75 %–95 % [1]*. E. coli* is known to cause meningitis and gastrointestinal diseases in vertebrates, including humans, in addition to a prevalence of UTIs.

Biochemical agents, such as antibiotics and sanitizers, were the primary means of controlling various bacterial pathogenesis and preventing bacterial reproduction in many sectors. However, bacterial multi-drug resistance was the outcome of the imprudent application of these chemical agents. Antibiotic resistance in *E. coli* isolates is also becoming more common [2], and antibiotic resistance has even been linked to the formation of new strains of resistant bacteria [3]. It is imperative to consider alternate treatments due to the rising prevalence of *E. coli*, which is resistant to many drugs [4]. Bacteriophages are regarded as a viable substitute for therapeutic applications [5,6].

Frederick Twort and Felix d'Herelle separately identified bacteriophages in cell cultures of *Staphylococcus aureus* and *Shigella*, respectively, in 1915 and 1917 [7]. D'Herelle employed phages for the first time in medicine in 1919. Despite encouraging outcomes, the discovery of antibiotics in 1940 led to a decline in researchers' interest in using bacteriophages to treat human diseases because of conflicting public views [8]. However, research on the use of bacteriophages has increased in various sectors such as clinics, industry, and environment management in the modern era.

Only particular prokaryote species, or even strains within the same species, are susceptible to phage infection. Bacteriophages can attach themselves to the surface of microorganisms by the use of proteins and other supporting structures. They can then inject their genetic material into the host and exhibit various infection cycles, such as lytic, lysogenic, pseudo-lysogenic, or chronic cycles. Phages exhibiting a lytic cycle of infection may be employed as preferred biocontrol agents. In this cycle, bacteriophages cause cellular lysis by injecting their genetic material into the cell to create new virus particles [6].

Significant modifications were made to the taxonomy of bacterial and archaeal viruses by the International Committee on Taxonomy of Viruses (ICTV). To group all-tailed bacterial and archaeal phage viruses with icosahedral capsids and a double-stranded DNA genome, the order *Caudovirales* was replaced by the class *Caudoviricetes*, and the families *Myoviridae*, *Siphoviridae*, and *Podoviridae* were abolished. This was the most significant change in phage classification to date [9].

The bacteriophage genome exists as one of the four possible forms of nucleic acids (ssDNA, dsDNA, ssRNA, and dsRNA) [10,11]. Phages differ greatly in their genetic makeup: their genome lengths range from 3405 bp to 497513 bp, their gene densities from 0.29 to 1.36, and the number of encoded proteins they contain from 1 to 675.

Bacteriophage genomes account for only about 31.6 % of the viral genomes in the database unique to the bacterium and archaea domains. The barrier to phage functional genomic research is bacteriophage DNA sequencing, which remains challenging despite introducing new sequencing tools [12]. Pure phage genomic material, PCR amplification, and the genetic material's complexity owing to intrinsic features like methylation bases and repetition zones, which are inherently challenging to sequence and organize, are the primary challenges [13].

From a technological perspective, bacteriophage sequencing is crucial for any functional genomics investigation. One of the most comprehensive methods for studying phage-encoded proteins is genome sequencing; nevertheless, genetic material only displays potentially expected proteins; it does not demonstrate how each of these proteins is expressed during host infection [14,15]. To fully comprehend phage-bacteria interactions, additional omics techniques such as transcriptomics, proteomics, and metabolomics could be employed in conjunction with phage genome sequencing [[16], [17], [18]].

Achieving a high enough coverage of phages of clinically relevant diseases to address future demand for therapeutic phages requires ongoing phage isolation and characterization due to the growing interest in phage therapy and the normally fairly narrow host range of phages. Thus, the study of phages and their genomes is intrinsically valuable to further our understanding of ecology, evolution, molecular biology, pathogenicity caused by bacteria, biotechnology, and health. Developing an understanding of phage genomes will undoubtedly open up possibilities for converting phages and unique phage proteins into effective biotechnological and medicinal instruments [19,20]. The genomes of diarrheagenic *E. coli* strain phages isolated from rivers, dairy farm sewage, and hospital fluid wastes were sequenced in this work. The six diarrheagenic *E. coli* strains used for isolation include enteropathogenic *E. coli* (EPEC), enterohaemorrhagic *E. coli* (EHEC/O157), enteroinvasive *E. coli* (EIEC), enteroaggregative *E. coli* (EAEC), enterotoxigenic *E. coli* (ETEC), and Shiga toxin-generating *E. coli* (Non-O157 STEC). Some phage-critical genes were identified from sequence data through comparative genome analysis with other sequenced *E. coli* genomes. This study provides researchers with genomic data on a lytic *E. coli* phage, which they can use for sequence comparison and evolutionary relationship analysis. The scientific community can also utilize phage genome sequencing data to screen for and identify new phage-based antibacterial treatments.

## 2 Materials and methods

### 2.1 Phage isolates

The lytic *E. coli* bacteriophages were isolated from various sources such as rivers, dairy farm sewages, and hospital fluid wastes in Addis Ababa, Ethiopia. These seven phages were lytic for five diarrheagenic *E. coli* strains (Table 1). Bacteriophage isolates were characterized morphologically, biologically, and molecularly using PCR and agarose gel electrophoresis (https://doi.org/10.21203/rs.3.rs-3653371/v2).

**Table 1:** Bacteriophage isolates used for genome sequence and analysis.

No	Phage isolates	Sample sources for isolation	*E. coli* host strains
1	EH-B-A (A1)	River	Enterohaemorrhagic *E. coli* (EHEC-O157)
2	EH-SD-TH	Hospital waste- Sediment	Enterohaemorrhagic *E. coli* (EHEC-O157)
3	EP-M-A	River	Enteropathogenic *E. coli* (EPEC)
4	EP-B-K (E2)	River	Enteropathogenic *E. coli* (EPEC)
5	EI-SP-GF	Superficial of dairy farm	Enteroinvasive *E. coli* (EIEC)
6	ET-SD-TH	Hospital waste- Sediment	Enterotoxigenic *E. coli* (ETEC)
7	ST-T-K	River	Shiga toxin-generating *E. coli* (STEC)

### 2.2 DNA extraction and quality analysis

To extract DNA from bacteriophages, the phenol-chloroform organic DNA extraction method was used, followed by an ethanol precipitation step. Using a Nanodrop spectrophotometer, the extracted DNA's quantity and purity have been determined. By performing a comparative DNA extraction using DNase, RNase, SDS, and Proteinase K treatment at various levels, the quality concerning host DNA contamination was evaluated. The quality and quantity comparative analysis was validated by agarose gel electrophoresis. The initial DNA concentration of the phages EP-M-A, EP-B-K, E2, EI-SP-GF, ET-SD-TH, and ST-T-K was 110.2, 129.9, 70.6, 387, 751.3 ng/50ul, respectively, with quality ranging from 1.8 to 2. Sequencing was done using the generated, evaluated, and quantified DNA extract.

### 2.3 Sequencing and assembly

#### 2.3.1 Phage genome sequencing

Phage lysate DNA samples were sent to the Armauer Hansen Research Institute (AHRI), Addis Ababa, Ethiopia for whole phage genome sequencing using an Illumina NextSeq 500/550 sequencer with 2x150 bp read length. Ten (10 μl) of the DNA was used for each sample for the whole genome library preparation with a modified Illumina COVIDseq RUO kit. The kit was modified by using phage-specific primers instead of viral RNA primers for amplification, employing adapters compatible with phage libraries, aligning the sequencing strategy with phage library goals, and adjusting the quality control steps for sequencing and functional analysis. In short, the DNA was enzymatically fragmented and tagmented simultaneously. The tagmented DNA was purified and amplified with a limited PCR cycle for the addition of indexes and amplification. The concentration of the libraries was measured using a Qubit HS assay kit. The concentrations are listed in Table 2. The libraries were finally loaded on the sequencer targeting 80x depth coverage. The resulting paired-end reads were obtained in the FASTQ format.

**Table 2:** The concentration of phage DNA library prepared for whole genome sequencing.

No	Sample ID	Phage isolates	DNA Concentration(ng/μl)
1	S41	EH-B-A (A1)	0.106
2	S42	EH-SD-TH	0.152
3	S43	EP-M-A	1.950
4	S44	EP-B-K (E2)	3.200
5	S45	EI-SP-GF	2.900
6	S46	ET-SD-TH	0.152
7	S47	ST-T-K	0.206

#### 2.3.2 Sequence assembly and consensus generation

In this study, a comprehensive analysis workflow was used for generating a high-quality consensus phage genome from Illumina sequencing data. The workflow involved several crucial steps designed to ensure accurate assembly and error detection and correction. Widely adopted bioinformatics tools such as FastQC, BBMap package, SPAdes, BWA, Sam tools, and Pilon were utilized to perform the necessary data processing and analysis. The quality of raw reads was checked initially using FastQC, and the bbduk.sh script from the BBMap package was employed to perform adapter trimming and quality filtering. This was followed by genome assembly using SPAdes version 2.0.0 on a Linux environment with filtering parameters of k-mer size, coverage threshold, contig length, and error corrections. From the resulting assembly, the longest contig was selected as the representative genome sequence. To prepare the longest contig for read alignment, it was indexed using the Burrows-Wheeler Aligner (BWA). Paired-end reads were then aligned to the longest contig using BWA's mem algorithm, and the resulting alignment was stored in a Sequence Alignment/Map (SAM) file. To facilitate downstream analysis, the SAM file was sorted by genomic coordinates using Sam tools, generating a sorted binary alignment map (BAM) file. Subsequently, an index file (bai) was created for the sorted BAM file using Sam tools. The sorted and indexed BAM file was then subjected to error detection and correction using Pilon, which utilizes the alignment information to identify and correct errors, including SNPs, indels, and gaps, resulting in an improved consensus phage genome. The corrected genome assembly was stored in a FASTA file and used for downstream analysis [21,22]. The variant calling and a consensus sequence were generated using Ivar tools.

### 2.4 Phage genome sequence analysis

The entire genome sequences were queried against the viruses (taxid:10239) nucleotide collection (nr/nt) using NCBI blastn and default settings (http://www.ncbi.nlm.nih.gov/genome). In addition, Genome Detective, ViPTree (http://www.genome.jp.viptree/), and VICTOR (http://ggdc.dsmz.de/victor.php) comprehensive web-based software were used to determine genome size and to classify phage genomes using sequence-derived taxonomic features to determine whether phages belong to the lytic phages class of *Caudoviricetes* [[23], [24], [25]]*.* RAST (http://rast.nmpdr.org/) online annotation server was used to annotate the whole genome of phage, and Genome Detective web-based software was used for the identification of CDSs and initial annotation of the phage genomes, including identification of the phage terminase large subunit, major capsid proteins, and phage lytic enzymes. The GeneMarkS and GC content calculators were used to determine the G + C content of the phages. The number of tRNAs was predicted using web-based software called tRNAscan-SE 2.0 (http://trna.ucsc.edu/tRNAscan-SE/). Using the Virulence Factor Database (VFDB), CRISPR Finder, and ResFinder, the virulence determinants, the CRISPR-like system, lysogeny, and the genes implicated in antibiotic resistance were identified [[26], [27], [28]]. The genome map of five phage isolates was created by using the Proksee genome analysis software [29].

The evolutionary relationship among five phage isolates was carried out by the VICTOR web service (https://victor.dsmz.de). All pairwise comparisons of the nucleotide sequences were conducted using the Genome-BLAST Distance Phylogeny (GBDP) method [30] under settings recommended for prokaryotic viruses [25]. The resulting intergenomic distances were used to infer a balanced minimum evolution tree with branch support via FASTME, including SPR postprocessing [31] for each of formulas D0, D4, and D6, respectively. Branch support was inferred from 100 pseudo-bootstrap replicates each. Trees were rooted at the midpoint [32] and visualized with ggtree [33]. Taxon boundaries at the species, genus, and family level were estimated with the OPTSIL program [34], the recommended clustering thresholds [25], and an F value (fraction of links required for cluster fusion) of 0.5 [35].

To ascertain the diversity of phage genomes and the evolutionary connections between phages, multiple sequence alignment was carried out using ClustalW, and a phylogenetic tree was built using the neighbor-joining and MEGA11 software methods. Major capsid protein and the conserved gene were utilized as phylogenetic phylo-markers for the variety and evolutionary relationship of each phage isolate. Reference sequences used in the analysis were obtained from the GenBank database. Phylogenetic trees were supported statistically by bootstrapping with 1000 replicates. Homologs were identified in the NCBI GENE database using the nucleotides as queries. The accession numbers of the viruses used in the alignments and phylogenetic analyses are listed on the trees.

The raw sequence data was submitted to the NCBI database as a sequence read archive (SRA) with PRJNA1006193 bio-project identifier and SAMN37015700 to SAMN37015706 bio-sample identifier. The quality control assays confirmed and assembled five phage genome sequences were submitted to GenBank with accession numbers from SRR25691062 to SRR25691068.

## 3 Results

### 3.1 Whole genome sequence analysis

To examine the genomic structures and the potential diversity of the genomes, the seven potent newly isolated bacteriophage genome sequences were determined, and genomic comparison was conducted. The good quality of raw sequence data for all phages was ensured according to FastQC parameters. Therefore, the sequences of phage isolates EH-B-A, A1, and EH-SD-TH were not further analyzed due to low-quality FastQC results for consensus sequence generation. As a result, five sequences were further analyzed and submitted to GenBank with accession numbers from OR992643_-_OR992647. Genome sequencing of the bacteriophages showed that all the bacteriophages have a linear double-stranded DNA genome. Phage genomes ranged in size from 24,264 to 136,204 bp, with a GC content between 44 and 55 % (Table 3, Table 4). According to the comprehensive genome analysis, the completeness of the genome indicated that all phage isolates were intact except for ET-SD-TH, which had a questionable genome. Aligned in the NCBI database, BLASTn analysis showed that the phage isolate EP-M-A had the highest genome similarity with Escherichia phage ZCEC5 (GenBank: NC-073321.1), with a nucleotide similarity of 91.81 % and genome coverage of 76 %. EP-B-K, E2, EI-SP-GF, ET-SD-TH, and ST-T-K had the highest genome similarity with Escherichia phage K1G (GenBank: NC-027993.1), Escherichia phage slur16 (GenBank: NC-028248.1), Escherichia phage vB_EcoM_ECO1230-10 (GenBank: NC-027995.1) and Escherichia phage vB_EcoS-101114BS4 (GenBank: NC-073061.1), respectively.

**Table 3:** The genome characteristics of phage DNA sequences.

Samples	GenBank Accessions	No raw reads	No trimmed reads	No contigs	Genome size (bp)	Subfamily	Genera
EH-B-A, A1	SRR25691062	1095	398	55	–	*-*	*-*
EH-SD-TH	SRR25691063	4329	1880	267	–	*-*	*-*
EP-M-A	SRR25691064	534845	276179	217	24264	*Guernseyvirinae*	*Kagunavirus*
EP-B-K, E2	SRR25691065	803828	377929	40	43553	*Guernseyvirinae*	*Kagunavirus*
EI-SP-GF	SRR25691066	624628	325380	114	136204	*Vequintavirinae*	*Vequintavirus*
ET-SD-TH	SRR25691067	10079	5024	511	33119	–	*Jilinvirus*
ST-T-K	SRR25691068	53297	22236	31	28657	–	*Dhillonvirus*

**Table 4:** Genome detective and RAST-based annotations of the phage genome.

Phages	No CDS	Functional proteins	No tRNA	GC content (%)	NT (%) Identity	AA (%) Identity	Alignment score	Reference phage
EP-M-A	33	23	No	52	91.8	96.1	119175	Escherichia phage ZCEC5 (taxon:2530021)
EP-B-K (E2)	77	50	No	51	91.35	96.23	108109	Escherichia phage K1G (taxon:698486)
EI-SP-GF	218	169	5	44	94.3	97.2	194829	Escherichia phage slur16 (taxon:1720495)
ET-SD-TH	41	8	No	53	88.1	88.4	76790	Escherichia phage vB_EcoM-ep3 (taxon:1541883)
ST-T-K	37	27	No	55	94.4	96.9	153955	Escherichia phage vB_EcoS-101114BS4: (taxon:2865793)

Note: CDS = coding sequences; tRNA = transfer ribonucleic acid.

The taxonomic classification of the 5 isolated potent coliphages was performed by multiple WGS genome comparisons (http://www.ncbi.nlm.nih.gov/genome/viruses/) and phage DB using comprehensive analysis. All phages were classified under the class *Caudoviricetes*. These coliphages included 2 (60 %) *Guernseyvirinae E. coli* phages, and the remaining 40 % included 1 *Vequintavirinae* and 2 subfamily unclassified *E. coli* phages. According to ICTV guidelines, the phage genus was predicted based on genome similarity. The results are shown in Table 3. EP-M-A and EP-B-K, E2 phage isolates belong to the genus *Kagunavirus.*

The phage EI-SP-GF was classified under the genus *Vequintavirus*, while ET-SD-TH and ST-T-K phage isolates belonged to the genera *Jilinvirus* and *Dhillonvirus*, respectively.

As indicated in Table 4, 33–218 putative CDSs were discovered for the *E. coli* phages using both automatic and manual annotation. All the coliphage genome sequences had CDSs that encoded the phage terminase small subunit, DNA polymerase, phage terminase big subunit, the phage lysis enzyme, and the phage capsid and tail proteins. In addition, tiny terminase components, tail fiber, baseplate spike proteins, and phage DNA polymerase were found in the majority of the phage genomes. One to three CDSs for the tail and capsid proteins of each phage were discovered. None of the phage genomes contained any known acquired resistance or virulence genes. The updated tRNAscan-SE-based predictions of tRNAs indicated that EI-SP-GF had 5 tRNAs. The remaining phage isolates have no tRNA.

**Fig. 1:**
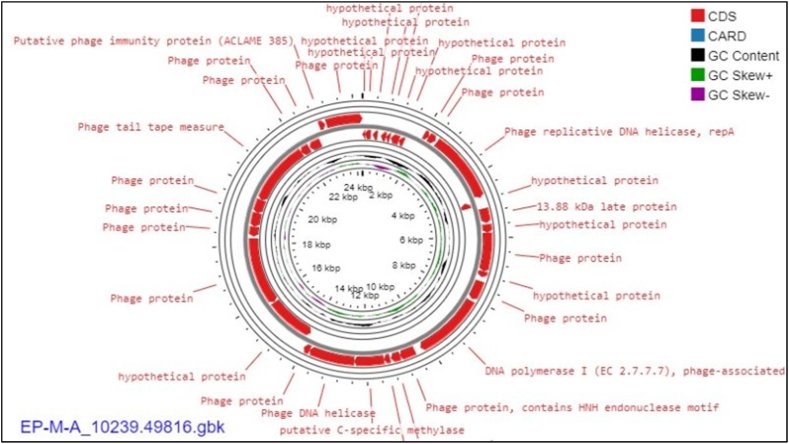
The circular genome map view of the linear sequence EP-M-A phage isolate: The number on the innermost circle indicates the base pair's position, and the genome is 24264 base pairs long. The bold red colored circle corresponds to distinct functions clockwise for the forward read box and counterclockwise for the back read box. The outermost ring reflects the 33 CDS and its function encoded by the gene. The colored circle in the middle shows the GC skew (G−C (pink)/G + C (green), while the black circle in the middle represents the GC content outwards, indicating larger than the average GC content compared with the complete genome, and inwards suggests the reverse. Towards the outside, it shows >0, and inward, it shows <0. A CARD with a blue color in the legend indicates the resistance gene identifier used in the analysis with no gene found in the map. (For interpretation of the references to color in this figure legend, the reader is referred to the Web version of this article.)

For the EP-M-A phage isolate, 17 (51.5 %) of the 33 predicted CDSs were discovered to be present in the direct strand. In contrast, the remaining CDSs were detected in the complementary strand (Fig. 1). Ten CDSs (30.3 %) were projected to encode putative proteins, while twenty-three CDSs (69.7 %) were expected to encode functional proteins. The fundamental phage-related functions of DNA replication/modification, packing protein, a structural protein, metabolism/regulation, and host lysis were found to be attributed to the CDSs. This phage's genome lacked genes encoding for the CRISPR-like system, virulence factors, toxins, antibiotic resistance, or hallmarks of temperate phages.

**Fig. 2:**
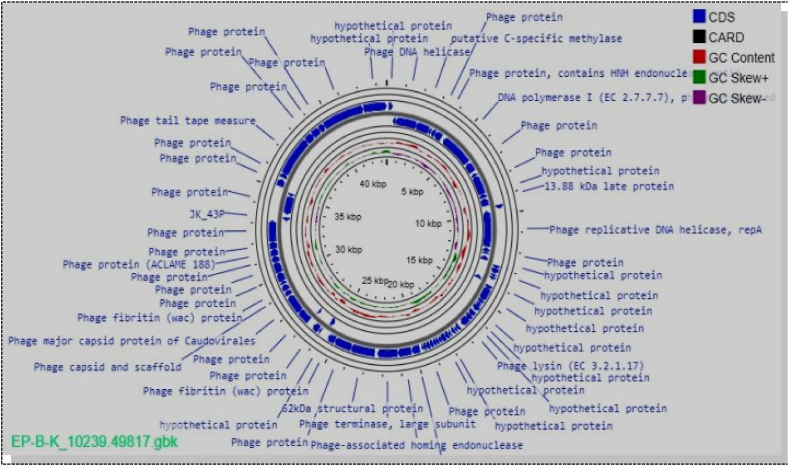
The circular genome map view of the linear sequence EP-B-K phage isolate: The number on the innermost circle indicates the base pair's position, and the genome is 43553 base pairs long. The bold blue colored circle corresponds to distinct functions clockwise for the forward read box and counterclockwise for the back read box. The outermost ring reflects the 77 CDS and its function encoded by the genome. The colored circle in the middle shows the GC skew (G−C (pink)/G + C (green), while the red circle in the middle represents the GC content outwards, indicating larger than the average GC content compared with the complete genome, and inwards suggests the reverse. Towards the outside, it shows >0, and inward, it shows <0.A CARD with a black color in the legend indicates the resistance gene identifier used in the analysis with no gene found in the map. (For interpretation of the references to color in this figure legend, the reader is referred to the Web version of this article.)

There were 77 putative coding sequences (CDSs) encoded in the whole genome of EP-B-K phage isolates. Of them, there were 27 (35.1 %) CDSs with unknown (speculative) functions and 50 (64.9 %) CDSs having identified functional proteins. It was discovered that there were 27 (28.6 %) in the lagging strand and 55 (71.4 %) in the leading strand (Fig. 2). The identified functional proteins include those that are necessary for phage replication, packaging, metabolism, and host lysis enzyme.

**Fig. 3:**
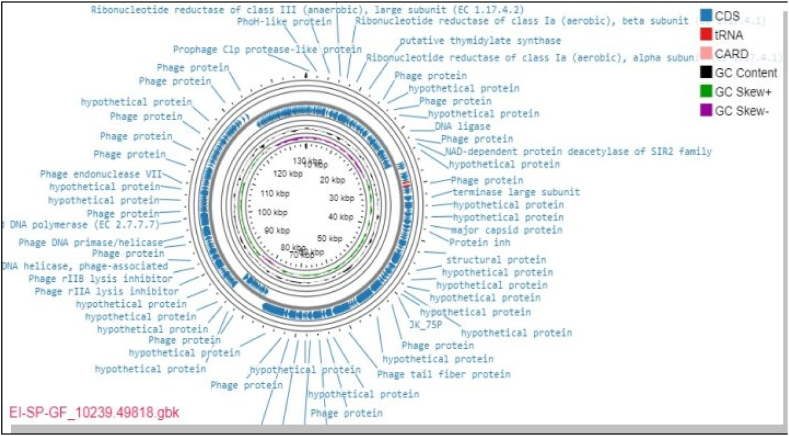
The circular genome map view of the linear sequence EI-SP-GF phage isolate: The number on the innermost circle indicates the base pair's position, and the genome is 136204 base pairs long. The bold dark blue colored circle corresponds to distinct functions clockwise for the forward read box and counterclockwise for the back read box. The outermost ring reflects the 218 CDS and its function encoded by the genome. The colored circle in the middle shows the GC skew (G−C (pink)/G + C (green), while the black circle in the middle represents the GC content outwards, indicating larger than the average GC content compared with the complete genome, and inwards suggests the reverse. Towards the outside, it shows >0, and inward, it shows <0. A CARD with a rose color in the legend indicates the resistance gene identifier used in the analysis with no gene found in the map. The red-colored CDS region in the map represents tRNA in the genome of this phage isolate. (For interpretation of the references to color in this figure legend, the reader is referred to the Web version of this article.)

The entire circular genomic map of the phage EI-SP-GF is demonstrated in Fig. 3. In the entire genome, 218 CDSs were predicted by the genome annotation study, of which 169 (77.5 %) encode functional proteins and 49 (22.5 %) hypothetical proteins. Of the CDSs, 126 (57.8 %) were discovered in the positive strand and 92 (42.2 %) in the negative strand. The genome of this phage was projected to have five tRNAs: tRNA-Arg-TCT, tRNA-Tyr-GTA, tRNA-Thr-TG, tRNA-Met-CAT, and tRNA-Pro-TGG.

**Fig. 4:**
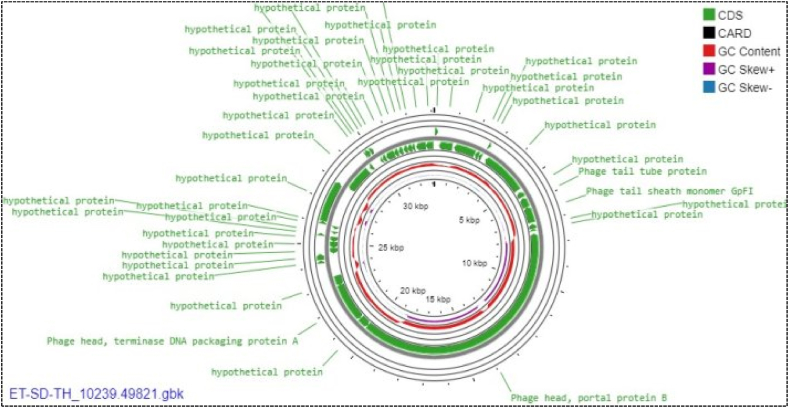
The circular genome map view of the linear sequence ET-SD-TH phage isolate: The number on the innermost circle indicates the base pair's position, and the genome is 33119 base pairs long. The bold green colored circle corresponds to distinct functions clockwise for the forward read box and counterclockwise for the back read box. The outermost ring reflects the 41 CDS and its function encoded by the genome. The colored circle in the middle shows the GC skew (G−C (dark blue)/G + C (pink), while the red circle in the middle represents the GC content outwards, indicating larger than the average GC content compared with the complete genome, and inwards suggests the reverse. Towards the outside, it shows >0, and inward, it shows <0. A CARD with a black color in the legend indicates the resistance gene identifier used in the analysis with no gene found in the map. (For interpretation of the references to color in this figure legend, the reader is referred to the Web version of this article.)

According to the ET-SD-TH phage isolate's whole genome map, 41 CDSs in all were predicted by utilizing the RAST and PHASTER software programs. Of them, 33 (80.5 %) CDSs were encoded for putative proteins, and only 8 (19.5 %) CDSs were shown to be functional proteins. Among these 41 putative genes, 10 were located in the forward strand and 31 in the complementary strand (Fig. 4). Fortunately, no genes encoding suspected poisons or resistance to antibiotics were found in the phage genome.

**Fig. 5:**
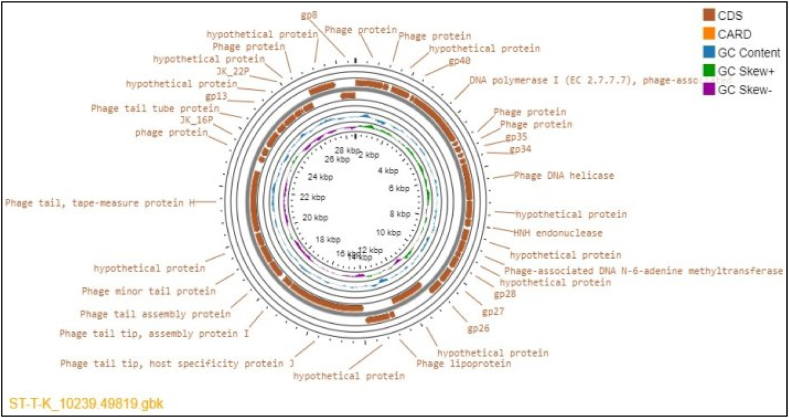
The circular genome map view of the linear sequence ST-T-K phage isolate: The number on the innermost circle indicates the base pair's position, and the genome is 28657 base pairs long. The bold dark red colored circle corresponds to distinct functions clockwise for the forward read box and counterclockwise for the back read box. The outermost ring reflects the 37 CDS and its function encoded by the genome. The colored circle in the middle shows the GC skew (G−C (pink)/G + C (green), while the blue circle in the middle represents the GC content outwards, indicating larger than the average GC content compared with the complete genome, and inwards suggests the reverse. Towards the outside, it shows >0, and inward, it shows <0. A CARD with an orange color in the legend indicates the resistance gene identifier used in the analysis with no gene found in the map. (For interpretation of the references to color in this figure legend, the reader is referred to the Web version of this article.)

The entire genome analysis of the ST-T-K phage isolate yielded 37 CDSs, of which 27 (73 %) encoded functional proteins and 10 (27 %) putative proteins. Of these, the forward strand had 21 CDSs, while the reverse strand contained the remaining CDSs (Fig. 5). Using the tRNAScan software, no tRNA gene was discovered. The general genome properties of coliphage isolate ST-T-K were gathered in Table 3, Table 4

### 3.2 Phylogenetic analysis

**Fig. 6:**
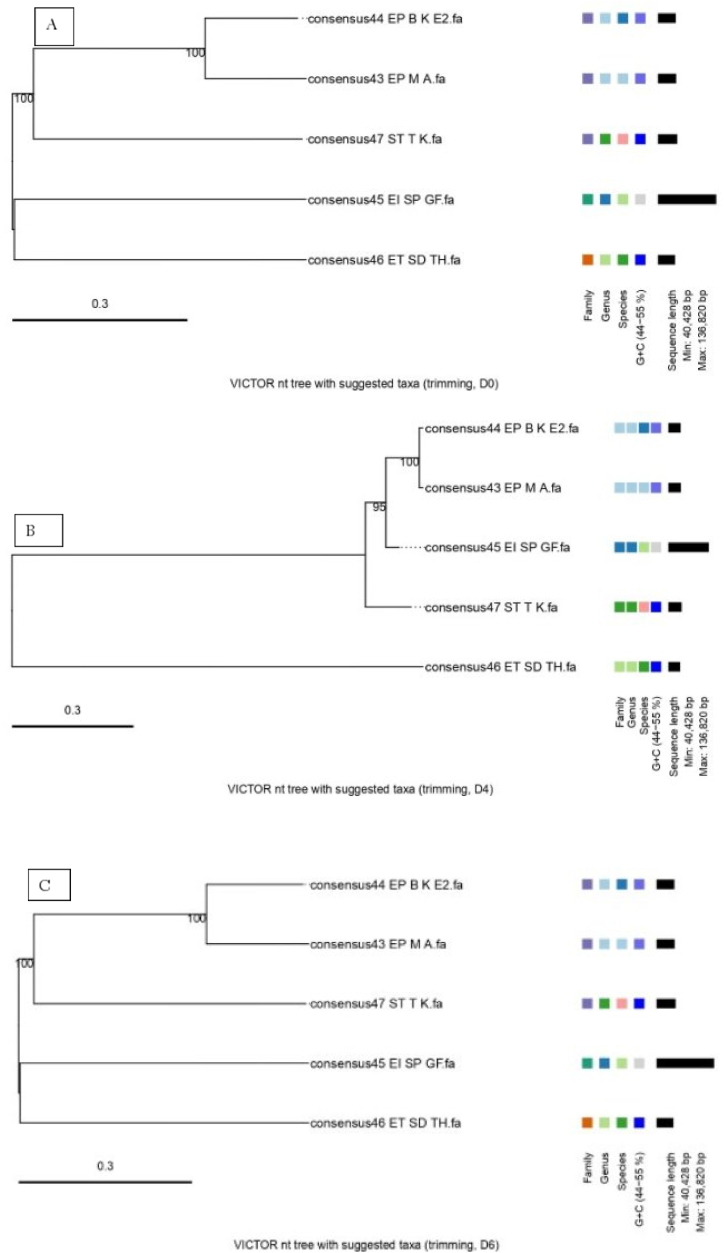
Phylogenetic tree among phage isolates constructed by using the sequence alignment of the Victor web service: A) Victor nt tree with suggested taxa (trimming, D0) B) VICTOR nt tree with suggested taxa (trimming, D4) C) Victor nt tree with suggested taxa (trimming, D6). A bootstrap value of 95 and 100 in the branch of the tree indicates the level of support in the branch that implies a 95 % and 100 % inferred relationship to be accurate.

Fig. 6 shows the phylogenomic GBDP trees inferred using formulas D0, D4, and D6 and yielding average support of 100 %, 97 %, and 100 %, respectively. The numbers above branches are GBDP pseudo-bootstrap support values from 100 replications. The branch lengths of the resulting VICTOR trees are scaled in terms of the respective distance formula used. The OPTSIL clustering yielded five (D0), five (D4), and five (D6) species clusters, respectively (Fig. 6A and B & C). At the genus level, four (D0), four (D4), and four (D6) clusters resulted, respectively. The number of clusters determined at the family level was three (D0), four (D4), and three (D6), respectively.

In phylogenetic trees of the relation among phage isolates, EP-M-A clustered together and in the same clade with phage EP-B-K, E2 with 100 % bootstrap values as both phages were in the same genus of *Kagunavirus*. These two phages isolate again showed a 100 % evolutionarily relationship with phage isolate ST-T-K. Phage isolate EI-SP-GF showed a relationship with the phage ET-SD-TH that was outgrouped from the relationship with other phages but within the same cluster.

To analyze the evolutionary relationship between phage isolates and other *Caudoviricete* phages, a phylogenetic tree was constructed based on the nucleotide sequences of the relatively conserved phage major capsid protein (MCP) phylomarker gene using the neighbor-joining (NJ) method. Constructing a major capsid protein-based phylogenetic tree of phages involves analyzing the genetic sequences of the major capsid protein gene from the database of different strains.

**Fig. 7:**
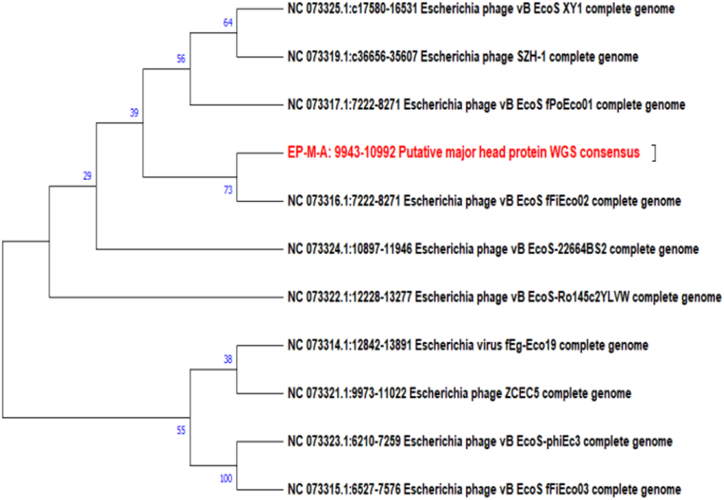
Phylogenetic tree of EP-M-A phage based on major capsid protein gene: The phylogenetic tree was constructed with 10 different phages under the genus *Kagunavirus.*

In the case of phage isolates EP-M-A and EP-B-K, E2 major capsid protein (MCP) gene search was limited to the genus *Kagunavirus.* The MCP gene sequence search for phage isolates EI-SP-GF, ET-SD-TH, and ST-T-K was limited to *Vequintavirinae*, *Jilinvirus,* and *Dhillonvirus*, respectively. Therefore, NCBI MCP gene sequences were retrieved from the database, the MCP gene was cut out from each phage isolate mapping with reference sequences, and multiple sequences were performed in MEGA11 software for tree construction. There were 10 NCBI search hit sequences obtained related to *Kagunavirus;* the accession numbers are indicated in the tree. The phylogenetic tree of phage isolates EP-M-A showed that phage EP-M-A and Escherichia phage vB EcoSfFiEco02 clustered onto a single branch with a 73 % bootstrap value, which strongly supports the inferred relations (Fig. 7). It was related by four phages by 39 bootstrap values, which means that the branch in question was supported in approximately 39 % of the resampled trees.

**Fig. 8:**
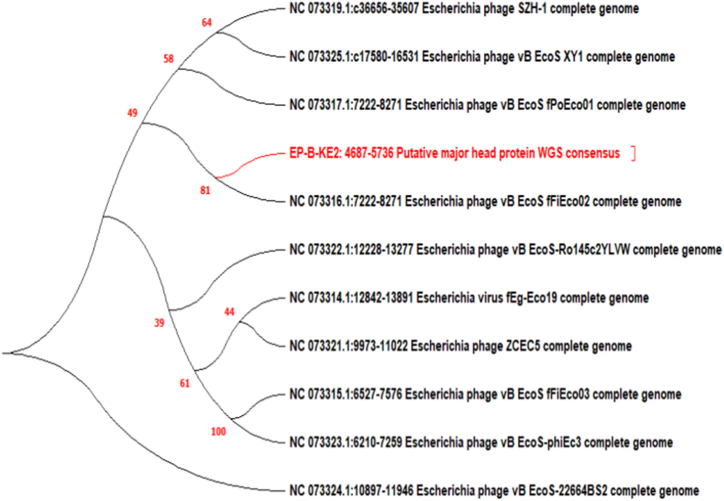
Phylogenetic tree of EP-B-K (E2) phage based on major capsid protein gene: The phylogenetic tree was constructed in relation to 10 different phages under the genus *Kagunavirus.*

The evolutionary relationship of phages EP-B-K (E2) showed that it was related to Escherichia phage vB EcoSfFiEco02 phage from the database with 81 % support of the grouping of taxa by bootstrap and 61 % support of the evolutionary relationship by two phages (Fig. 8).

**Fig. 9:**
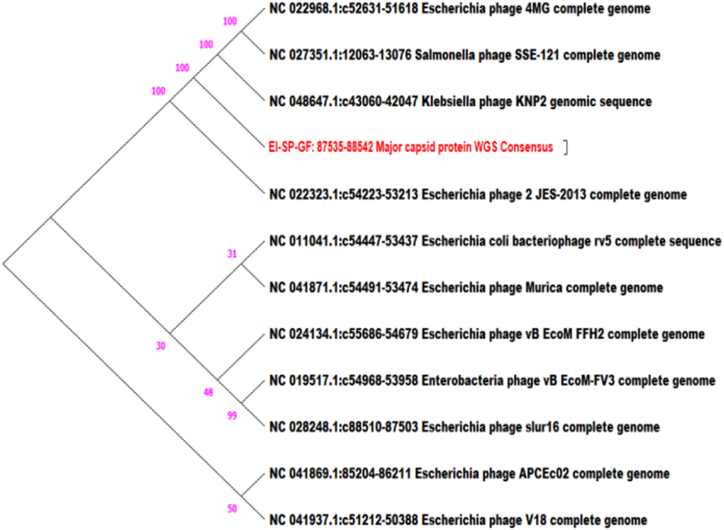
Phylogenetic tree of EI-SP-GF phage based on major capsid protein gene: The phylogenetic tree was constructed with 11 different phages under Subfamily *Vequintavirinae.*

The phage isolate EI-SP-GF was related 100 % with four phages including *Salmonella* and *Klebsiella* phages (Fig. 9).

**Fig. 10:**
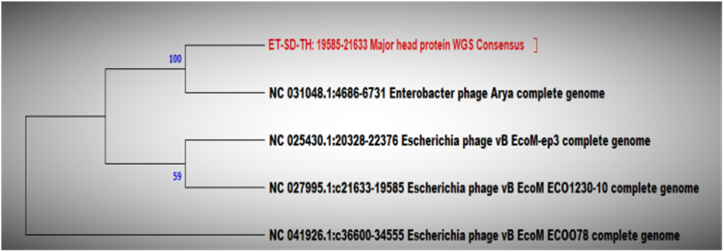
Phylogenetic tree of ET-SD-TH phage based on major capsid protein gene: The phylogenetic tree was constructed in relation to 4 different phages under the genus *Jilinvirus*.

**Fig. 11:**
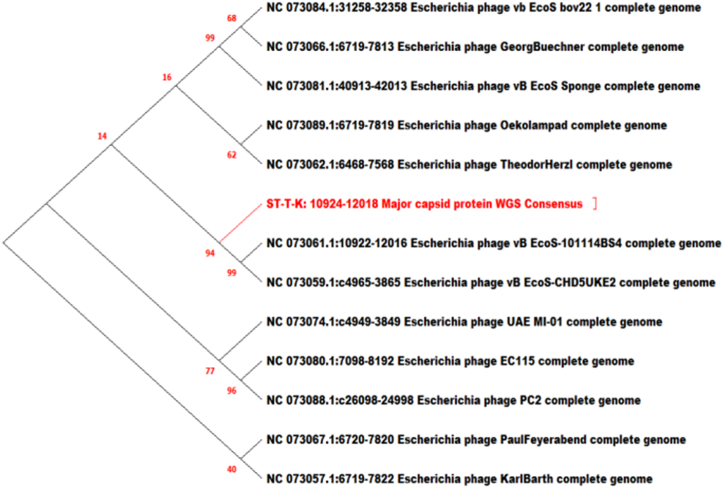
Phylogenetic tree of ST-T-K phage based on the major capsid protein gene: The phylogenetic tree was constructed with 12 different phages under the genus *Dhillonvirus.*

ET-SD-TH and ST-T-K were related 100 % with *Enterobacter* phage Arya and 94 % with two *E. coli* phages respectively (Fig. 10, Fig. 11).

## 4 Discussion

Multi-drug resistance of pathogenic *E. coli* has emerged recently as a result of the overuse of antibiotics, which is having a detrimental effect on food safety, human health, and the environment [36]. Researchers are currently looking for novel antibacterial treatments to address the growing problem of bacterial resistance. Phage treatment is one such remedy that is gaining popularity because of its bactericidal properties and bacterial host specificity. Isolating and genome-wide characterizing of lytic phages is crucial for the development of phage treatment against AMR bacterial infections. The genome architecture of lytic phages contains genes that encode multiple putative proteins with unclear functions. Therefore, genomic study can aid in both the taxonomic classification of phages and the identification of critical genes and suitable candidates for phage treatment [37].

In this study, seven diarrheagenic *E. coli* phages that isolated from rivers, dairy farms, and hospital fluid wastes, infecting six strains of *E. coli* that are resistant to many drugs. The study is consistent with the report by Refs. [38,39], which demonstrated the value of phage therapy in combating pathogenic *E. coli* that is resistant to currently available antibiotics. The phages' entire genomes were analyzed and characterized. Based on genomic studies, linear dsDNA genomes ranging in length from 24264 to 136204 bp, and with a GC content varying from 44 to 55 % were found in all phages. The phages that infect diarrheagenic *E. coli* were categorized as small because their genomes were less than 200 kbp in size. These results were in line with the earlier research conducted by Ref. [40], which demonstrated that other pathogenic *E. coli* O177 was infected by small phages.

These phages were categorized by BLASTn, Genom Detective, and PHASTER analysis into the class *Caudoviricetes* with subfamilies *Guernseyvirinae* and *Vequintavirinae*. Furthermore, diarrheagenic *E. coli* phages were also assigned to four genera, including *Kagunavirus* (EP-M-A and EP-B-K, E2), *Vequintavirus* (EI-SP-GF), *Jilinvirus* (ET-SD-TH), and *Dhillonvirus* (ST-T-K). It's interesting to note that the outcomes of the entire genome study indicated that all of the phages would be virulent, which qualified them for phage therapy.

In phage isolate ET-SD-TH, only 20 % of coding sequence regions (CDSs) code for putative and hypothetical proteins, whereas in the case of EP-M-A, EP-B-K, E2, EI-SP-GF, and ST-T-K, more than 90 % of CDSs code for proteins. The lower percentage of CDSs (20 %) coding for proteins in the ET-SD-TH isolate might be attributed to sequence low read numbers. This suggests that the sequencing depth or coverage for this isolate might be insufficient, resulting in a smaller fraction of identified CDSs. The higher percentage of CDSs coding for proteins in the EP-M-A, EP-B-K, E2, EI-SP-GF, and ST-T-K isolates (more than 90 %) indicates a greater level of genome completeness. These isolates likely have higher quality and depth of sequencing, which enables the identification of a larger proportion of functional protein-coding genes.

The phages of *E. coli* contain a large number of distinct genes that encode potential and functional proteins. Many of the CDSs identified in the phage genomes, most particularly ET-SD-TH, were hypothesized to be hypothetical proteins with enigmatic roles. Comparable findings have been documented for other different phages that infect pathogenic bacterial species [41,42]. This suggests that multiple genes with unknown functions are present in phage genomes. Therefore, research efforts must be focused on clarifying the actual roles of these putative proteins.

The presence of genes coding for phage DNA replication/modification (DNA polymerase I, DNA helicase, putative DNA cytosine methyltransferase C5, putative HNH endonuclease, DNA methyltransferase, DNA recombination nuclease inhibitor gamma), DNA synthesis and packaging (terminase large subunit, putative terminase small subunit), structural proteins (capsid and tail proteins), and host lysis (phage lysin, u-spin, putative holin-like class I protein, and putative holin-like class II protein) were among the intriguing findings. Two phage genomes (EP-B-K, E2, and EI-SP-GF) differed from other phages in that they have genes that encode tail fiber and baseplate tail spike proteins. The inclusion of tail fiber and tail spike proteins in a phage genome can improve its infection capabilities and host range because these proteins are essential for phage receptor recognition [43].

The tRNAscan-SE v. 2.0 analyses indicated that phage isolate EI-SP-GF had tRNAs in its genome, but the isolates EP-M-A, EP-B-K, E2, ET-SD-TH, and ST-T-K had no tRNAs. The absence of tRNA sequences in the phage genome implies that the phage is more reliant on the host cell's resources for translation and may have evolved to exploit the host's existing translational machinery. The presence of tRNAs in the phage genome suggests that the phage has adapted to replicate efficiently within the host cell by utilizing its translation machinery. In this study, the genome detective web-based and GeneMarkS-2 analysis showed that the genomes of all phage isolates do not contain sequences of genes encoding integrase, recombinase, repressors, or excisionase, which are the main markers of lysogenic viruses [44]. Therefore, the results indicated that these phages should be considered strictly lytic (virulent) phages, which is similar to the study by Ref. [45], which indicated the phage phiLLS's genome analysis shows that it is strictly lytic, meaning that it lacks genes linked to virulence factors and/or proteins that may be immunoreactive allergens.

To obtain a more global phylogenetic overview of the relationships between the different *E. coli* phage isolates, whole genome-based alignment was employed for tree construction against each other. The major capsid protein gene database sequences of the same genus as well as subfamily, particularly isolate EI-SP-GF, were obtained for the determination of the evolutionary relationship of each phage isolate with available database sequences. Bootstrapping, a resampling statistical technique, was used to assess the robustness of the inferred phylogenetic relationships. The resulting trees were compared to calculate the frequency at which a particular branch appears in the replicate trees. This frequency was expressed as a bootstrap value, which represents the statistical support for that branch. Higher bootstrap values (typically ranging from 70 to 100) indicate greater support for the branch [46].

They are constructed based on similarities and differences in genetic sequences, typically using multiple sequence alignment and evolutionary models. The phages EP-M-A and EP-B-K, E2 were clustered together, having 100 % support by bootstrapping. In phylogenetic analysis, bootstrap support is a measure of the statistical confidence or robustness of a particular branch or grouping in the tree. It is often represented as a percentage and indicates how often a particular grouping appears in replicate analyses of the data. A bootstrap value of 100 % suggests that in multiple iterations of the analysis, the sequences EP-M-A and EP-B-E2 consistently clustered together as a distinct group. This high bootstrap support indicates strong statistical confidence in the grouping or relationship between these two sequences. Therefore, based on the available information, it can be concluded that EP-M-A and EP-B-E2 are closely related and form a cluster in the phage phylogenetic tree, supported by a bootstrap value of 100 %, which is comparable with the study by Ref. [47], who constructed a phylogenetic tree using major capsid proteins, and many of the sequences from their phage genomes cluster together with high bootstrap support that defines clades.

The major capsid protein (MCP) gene was used for phylogenetic tree construction for each phage isolate to observe evolutionary relationships with existing database sequences. The MCP gene was the gold standard for the classification of the lytic phage family. An essential function of the major capsid protein (MCP) gene is to maintain the structure and function of bacteriophages, which are viruses that specifically infect and replicate within bacteria. The MCP gene encodes the major structural protein that forms the outer capsid of the phage, encompassing the viral genome and protecting it during infection. The MCP gene is highly conserved within a specific phage family, meaning that it displays a relatively low rate of mutation across different phage isolates within the same family. This conservation leads to utilizing the MCP gene for phylogenetic tree construction, which helps to elucidate the evolutionary relationships between different phage isolates [48,49]. The MCP gene database search was specified to the genus *Kagunavirus* for isolates EP-M-A and EP-B-K, E2 whereas in the case of EI-SP-GF specified to the family *Vequintavirinae, Jilinvirus* for ET-SD-TH, and *Dhillonvirus* for ST-TK to retrieve sequences from the database. Isolates EP-M-A and EP-B-K were classified within the genus *Kagunavirus*. This suggests that these isolates share significant similarities in their MCP gene sequences, indicating a close evolutionary relationship that is similar to the report by Ref. [50]. The isolate EI-SP-GF was classified within the family *Vequintavirinae*, a taxonomic family that encompasses a group of viruses showing similarities in their MCP gene sequences. The isolate ET-SD-TH was classified within the genus *Jilinvirus*, while the isolate ST-TK was classified within the genus *Dhillonvirus*. These genera represent distinct groups of viruses with shared MCP gene characteristics. All five phage isolates were closely linked to other phages, as demonstrated by both phylogenetic analyses based on the conserved MCP gene and whole-genome sequence alignment study. This implied that these phages had an intricate evolutionary relationship.

The NCBI Genome database only contains a small number of full phage genomes, even though bacteriophages are more widely distributed and varied. Specifically, there are extremely few reports on the diarrheagenic *E. coli* lytic phage family in the *E. coli* phage database. In addition, more updates and enhancements are needed for the phage virology database. In addition, there is a paucity of knowledge on the phage host's genome. The genome and functional annotation of bacteriophages remain mostly unexplored. Similarly, our study indicated that mostly annotated genes were encoded for hypothetical proteins. Therefore, it is recommended to use different experimental approaches such as protein-protein interactions, metabolomics and transcriptomics, and structural biology to investigate the function of those genes. Genome-wide characterization, lytic enzyme analysis, and in vivo profiling remain unexplored in this study, and future studies will give more insight into them.

Developing bacteriophages into microbial agents requires a thorough grasp of the phages. Whole genome sequencing is the most effective technique for determining the genetic background of phages, the underlying mechanisms that underlie phage-host interaction at the gene level, and a theoretical framework for phage genetic alteration.

## 5 Conclusion

The purpose of this work was to characterize and analyze the entire genome sequences of seven potential lytic phages that were isolated from hospital fluid effluent, dairy farms, and rivers. These phages were intended to combat different strains of multidrug-resistant diarrheagenic *E. coli* strains that are frequently associated with infections in humans and animals. The coliphages were shown to belong to the class *Caudoviricetes*. According to the whole genome sequence alignment and phylogenetic analysis, these phage isolates were closely related to *E. coli* and *Enterobacteria* phages in the database. Furthermore, the gene content and putative functions were determined from the genomes, which revealed the presence of phage protein genes and bacterial lysing enzymes. The genes responsible for lysogeny, virulence, toxins, and antibiotic resistance were absent from all phage isolates. Based on these features, the phages are suitable and promising candidates for therapy against strains of *E. coli* that are resistant to multiple drugs. Furthermore, the study expands on our comprehension of the genomic diversity of phages and provides useful basic information for further research on the interaction between phages and their hosts. With the use of this knowledge, it will be possible to decide whether to apply phage-based intervention in clinical settings following more genome characterization. Subsequent investigations into the role of phages in the regulation of antibiotic resistance will concentrate on assessing the phage's susceptibility to different Enterobacteriaceae family members. Aside from *E. coli*, isolated bacteriophages were not tested against any other pathogenic or non-pathogenic bacteria.

## CRediT authorship contribution statement

**Tamirat Salile Sada:** Writing – original draft, Methodology, Investigation, Formal analysis, Data curation, Conceptualization. **Dawit Hailu Alemayehu:** Visualization, Resources, Funding acquisition. **Kalkidan Melaku Tafese:** Visualization, Investigation. **Tesfaye Sisay Tessema:** Writing – review & editing, Supervision, Resources, Project administration, Funding acquisition.

## Availability of data and materials

The datasets used and/or analyzed during the current study are available from the corresponding author upon reasonable request till NCBI release.

## Ethics approval and consent to participate

There are no human or animal participants in this study.

## Consent for publication

“Not applicable” in this section.

## Funding

There has been no significant financial support for this work that could have influenced its outcome.

## Declaration of competing interest

The authors declare that they have no known competing financial interests or personal relationships that could have appeared to influence the work reported in this paper.

## List of abbreviations

E. coli: Escherichia coli
EAEC: Enteroaggregative *E. coli*
EHEC: Enterohaemorrhagic *E. coli*
EPEC: Enteropathogenic *E. coli*
ETEC: Enterotoxigenic *E. coli*
STEC: Shiga toxin-producing *E. coli*
UTI: Urinary tract infection
AMR: Antimicrobial resistance
CDS: Coding sequence
MCP: Major capsid protein
BWA: Burrow-wheeler alignment
BAM: Binary alignment map
BBMap: Big blue mutation analysis page
